# The emerging biology of muscle stem cells: Implications for cell-based therapies

**DOI:** 10.1002/bies.201200063

**Published:** 2012-08-06

**Authors:** C Florian Bentzinger, Yu Xin Wang, Julia von Maltzahn, Michael A Rudnicki

**Affiliations:** 1 The Sprott Centre for Stem Cell Research, Regenerative Medicine Program, Ottawa Hospital Research InstituteOttawa, Ontario, Canada; 2 Department of Cellular and Molecular Medicine, Faculty of Medicine, University of OttawaOttawa, Ontario, Canada

**Keywords:** myogenesis, PAX7, satellite cells, stem cells, therapy

## Abstract

Cell-based therapies for degenerative diseases of the musculature remain on the verge of feasibility. Myogenic cells are relatively abundant, accessible, and typically harbor significant proliferative potential ex vivo. However, their use for therapeutic intervention is limited due to several critical aspects of their complex biology. Recent insights based on mouse models have advanced our understanding of the molecular mechanisms controlling the function of myogenic progenitors significantly. Moreover, the discovery of atypical myogenic cell types with the ability to cross the blood-muscle barrier has opened exciting new therapeutic avenues. In this paper, we outline the major problems that are currently associated with the manipulation of myogenic cells and discuss promising strategies to overcome these obstacles.

## Introduction

Muscles all over the body are heterogeneous, differing in function, cellular composition and biochemical properties 1. The basic characteristics of different types of skeletal muscle are inherently determined according to its anatomic location, but can be influenced by changes in functional demand or by the metabolic state 2. This heterogeneity contributes to a variety of phenotypes associated with degenerative diseases of the muscular system 3. Most prominent are the muscular dystrophies. This group of diseases is largely caused by mutations in genes coding for proteins linking the extracellular matrix (ECM) to the muscle fiber membrane and further on to the contractile apparatus 4. Muscular dystrophies can affect distinct muscle groups and differ in severity from early lethality to mild forms with normal life expectancy 5. Because of the genetic basis of muscular dystrophies, viral gene therapy and cell-based approaches have been considered promising therapeutic strategies 6, 7. The absence of tumorigenicity and ability of myogenic progenitors to add their DNA to the syncitial muscle fibers by fusion makes these cells an ideal vector for genetic correction 8.

**Figure 1:**
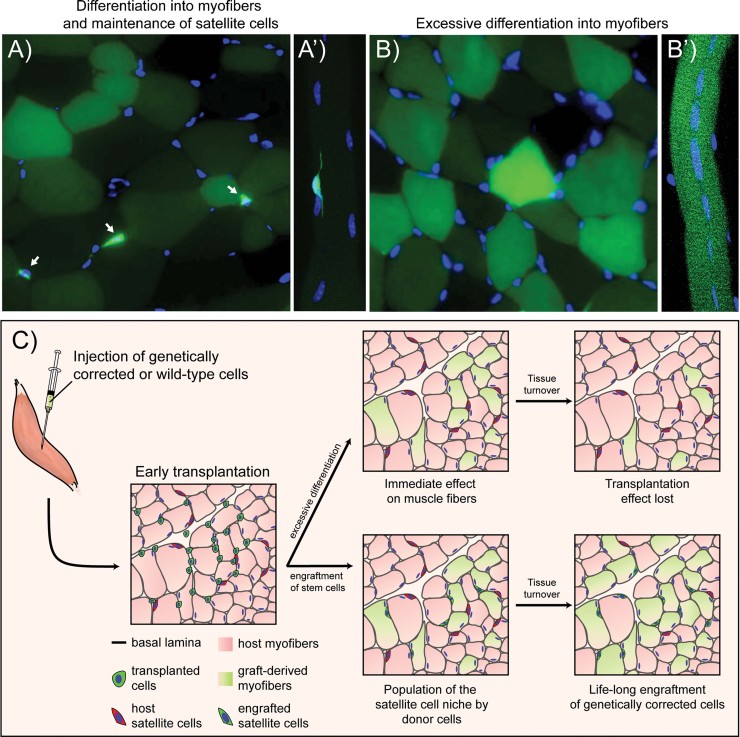
Transplantation of genetically corrected cells requires engraftment into the satellite cell compartment. Since myogenic precursors fuse with damaged myofibers to form a single syncytium, establishing a genetically-corrected stem cell compartment will lead to the long-term replacement of diseased tissue. A: Cross-section through the TA muscle showing GFP+ satellite cells (arrows) and myofibers. A′: A GFP+ satellite cell is observed on a single GFP− myofiber. In this case, GFP+ satellite cells will participate in future remodeling of muscle tissue and incorporate genetic corrections into host myofibers making them GFP+ as well. A graft of committed progenitors rather leads to excessive differentiation and will marginally engraft into the stem cell compartment. B: GFP is only found in myofibers but not satellite cells. B′: Micrograph of a GFP+ myofiber which is derived from GFP+ satellite cells that differentiated. Note that all fiber associated cells are GFP−. Although the establishment of genetically-corrected myofibers is the ultimate goal, without a stem cell population, the effects of these transplants are likely to diminish due to tissue turnover. C: Cartoon schematic of the possible long-term transplantation outcomes described above.

Unfortunately, a number of problems are associated with the sole genetic correction of muscle fibers. In healthy young muscle, the turnover of postmitotic muscle fibers is barely detectable 9. However, mutations leading to muscular dystrophy are thought to induce small tears in the sarcolemma of muscle fibers triggering their necrosis and apoptosis 3. As a consequence, muscle fibers in dystrophic muscles are constantly replaced by new regenerating fibers or scar-tissue 3. Immune cells which infiltrate de- and regenerating muscle can produce cytotoxic levels of nitric oxide and induce further plasma membrane damage through the release of myeloperoxidase 10–12. Moreover, the persistent inflammation which is characteristic for many forms of muscular dystrophy can provoke an excessive accumulation of ECM resulting in permanent fibrotic scar formation that impedes the differentiation of myogenic progenitors 13. Assuming that efficient anti-inflammatory and anti-fibrotic treatment is available, grafted cells could eventually establish genetically corrected muscle fibers that can withstand this cytotoxic and fibrotic environment. Nevertheless, there is evidence that muscle fibers turn over with aging, which would lead to a secondary loss of corrected fibers from the tissue 9, 14, 15. Other concerns are that cells that immediately fuse to fibers after transplantation would only lead to focal genetic correction around the injection site as opposed to a muscle-wide effect. Therefore, a strategy that sustainably replaces the self-renewing endogenous progenitor pool in a muscle-wide fashion with either genetically corrected or healthy donor cells would be more desirable than the transplantation of cells that are prone to focal irreversible differentiation (Fig. 1).

Satellite cells, the predominant myogenic cells in skeletal muscle, have a strong dependence on their niche consisting of specialized heparan sulfate rich microenvironment and adhesion molecules on the myofiber plasma membrane 16. In addition, satellite cells are always found in close proximity of blood vessels and their function can be modulated by other cell types such as fibroblasts, fibro/adipogenic progenitors (FAPs) and immune cells 17–19. Removal of satellite cells from their niche and expansion on cell culture dishes rapidly leads to commitment toward differentiation and converts satellite cells into a cell type that is commonly referred to as “myoblast” 20. Multiple studies in mice have demonstrated that satellite cells which have been converted into myoblasts through in vitro culture rapidly differentiate and cannot efficiently repopulate the satellite cell niche upon transplantation 21–23. Interestingly, recent studies with human myoblasts suggest that cultured cells can still give rise to satellite cells upon transplantation into irradiated mouse muscle 24. However, likely due to the limited availability of freshly isolated material, uncultured cells were not transplanted as a comparison. Reports of grafted single fibers, containing less than ten satellite cells, leading to muscle wide repopulation by donor cells, raise the question whether patients would profit more from transplantation of a few cells which are still associated with their niche as opposed to a large quantity of passaged myoblasts 25. Moreover, recent studies suggest the presence of a subpopulation of cells with characteristics of satellite cell progenitors within the satellite cell pool 26. Such satellite stem cells have been shown to self-renew and to have a superior capacity to repopulate the satellite cell niche upon transplantation without premature differentiation. Translational research focusing on the concept of satellite stem cells and their niche-addiction undoubtedly holds great therapeutic promise. In the following sections we will discuss key aspects of the biology of myogenic progenitors that is mostly based on mouse model systems and we will extrapolate their relevance for future stem cell therapy of diseased muscle.

## The muscle stem cell niche

**Figure 2:**
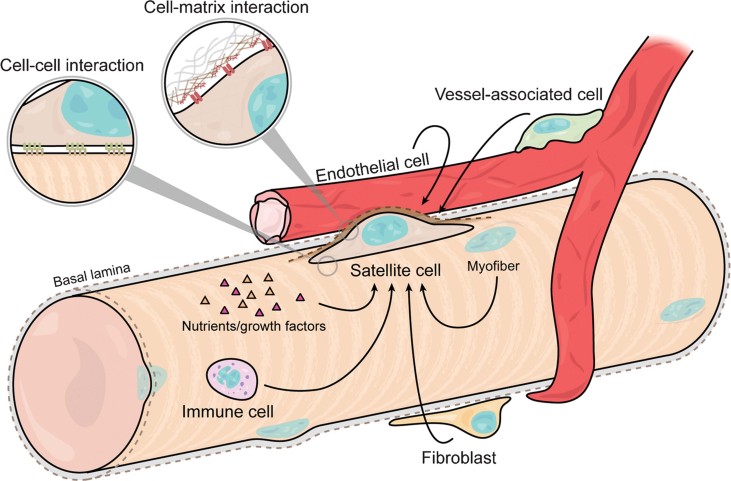
Representation of the satellite cell niche. Satellite cells reside within a specialized microenvironment, tightly packed between the ECM and their host myofibers. Cell-cell vs. cell-matrix interactions polarize the satellite cell niche in an apical-basal orientation and play a role in the determination of cell fate in asymmetric divisions. Paracrine interactions with various other cell-types (immune cells, fibroblasts, endothelial cells, vessel-associated cells, and the host myofiber) also modulate satellite cell behavior during homeostasis and regeneration.

The majority of muscle satellite cells spend the most of their lifetime quiescent in their niche (Fig. 2) 27, 28. Quiescent satellite cells have little cytoplasm and a condensed nucleus 28, 29. They sit flat on the muscle fiber membrane underneath a basement membrane composed of a complex ECM 29. Specialized adhesion molecules bolster the muscle fiber plasma membrane on which the satellite cell resides 16. Well-known amongst these are m-cadherin and the sialomucin CD34, the latter of which is downregulated in activated satellite cells 30. Satellite cells express high levels of membrane associated heparan sulfate proteoglycans (HSPG) such as Syndecan-3 (Sdc3) and Syndecan-4 (Sdc4) 31. HSPGs are able to sequester soluble ligands such as growth factors 32. Therefore, the local concentration of such factors in the HSPG-rich satellite cell niche can be substantially increased over the rest of the extracellular environment.

It appears that satellite cells also remodel their ECM microenvironment upon activation. While quiescent satellite cells express specific protease inhibitors, proliferating cells express high levels of matrix metalloproteinases (MMP) 33. It is plausible that upon muscle injury, local myogenic precursors have to become mobile and cross ECM barriers. In agreement with this idea, satellite cells which are activated on isolated single fibers in culture, can quickly leave their quiescence niche and melt through the ECM of the basal lamina 34, 35. Supporting this observation, satellite cells that were transplanted with an associated single fiber engraft throughout the host muscle, indicating that they can extensively migrate through the muscle ECM in vivo 25, 36. Moreover, studies using whole muscle grafts demonstrated that regenerating muscles might in fact receive myogenic precursors from neighboring muscle groups 37. Taken together, this suggests a model in which the satellite cell niche microenvironment is broken down or abandoned upon activation and satellite cells only depend on a specialized niche for their exit and return into quiescence. Alternatively, after leaving their niche, motile activated satellite cells might continue to autoregulate their ECM microenvironment. It is however, important to consider that it has never been formally proven that satellite cells cross the intact basal lamina during normal muscle regeneration in vivo.

The importance of the muscle fiber basal-lamina is illustrated by the severe phenotype of mice with mutations in the laminin alpha 2 chain 38. Such animals display a severe muscular dystrophy and a profoundly impaired regenerative capacity that is restored by re-establishment of a functional basal lamina 39. When plated on laminin myoblasts become polarized, elongated, and display increased locomotion 35. Moreover, activated satellite cells express high levels of the integrin alpha7-beta1 laminin receptor, which is required for their normal migration. This suggests that laminin mediated movement of satellite cells along the basal lamina sheet is an important process during muscle regeneration.

## Non-myogenic cell types in the satellite cell niche

An interesting scenario with respect to the satellite cell niche concept is that non-myogenic cell types could accompany activated satellite cells and contribute to the maintenance of their microenvironment. Fibroblasts which are well known to express high levels of ECM molecules and have no direct myogenic potential, are required for efficient muscle regeneration 40. Therefore, similar to other stem cell niches such as the ones found in the skin, the hematopoietic system, the central nervous system, or the intestine crypt, the satellite cell microenvironment is influenced by distinct tissue resident cell types 41. Whether fibroblasts engage in cell-cell contact, release tropic signaling molecules for satellite cells or whether they provide adhesive ECM substrates remains to be determined.

In 2005, Collins et al. demonstrated that transplantation of single fibers with their niche associated satellite cells leads to an outstandingly efficient engraftment into immunocompromised mdx recipient mice 25. Subsequently, the Olwin group modified this approach and isolated single muscle fibers, treated them with basic fibroblast growth factor (bFGF), suspended them in myotoxic solution and then transplanted them into non-immunocompromised wild-type mice 36. The authors reported that this kind of transplantation led to a highly efficient population of the satellite cell compartment as well as to a dramatic age-persistent increase in muscle size. These results strikingly illustrate that an intact niche allows for the maintenance and manipulation of satellite cells retaining a high stemness ex vivo. An interpretation of the outstanding engraftment efficiency reported by Hall et al. is that the bFGF in the culture medium altered the function of the fiber-associated satellite cells toward a more proliferative phenotype. However, other cell types such as fibroblasts that are attached to muscle fibers must also be taken into consideration. bFGF strongly promotes the proliferation and migration of ECM producing fibroblasts 42. The stimulation of these cells by the bFGF treatment could increase the availability of survival cues or preserves the structural microenvironment of satellite cells during transplantation. Moreover, bFGF can be sequestered on either cell surface heparan sulfate (HS) or matrix glycosaminoglycans. It is therefore also possible that exposure of single fibers to bFGF-rich culture medium saturates the ECM around the transplanted fibers. This could dramatically prolong its effect on the fiber-associated cells and thereby promote the engraftment of the transplanted satellite cells.

Next to fibroblasts, other non-myogenic cells, including endothelial cells and FAPs, have been implicated in the regulation of myogenesis 17, 43. In their niche, satellite cells are closely associated with blood vessels and the number of satellite cells in a given muscle type correlates positively with the abundance of capillaries 19. It has been proposed that vascular endothelial growth factor (VEGF) signaling from endothelial cells stimulates the proliferation of satellite cells during muscle regeneration while Angiopoietin 1 (Ang1) signaling from periendothelial cells, such as smooth muscle cells and fibroblastic cells, instructs their return to quiescence in later stages of myogenesis 43, 44.

Taken together, a variety of non-myogenic cell types contribute to the microenvironment of satellite cells and are likely to play a role in the preservation of their stem cell properties. This knowledge has important implications for future cell therapy. The concept of supportive cell types for the maintenance of stemness has long been applied in case of embryonic stem (ES) cells which are routinely cultured on a supportive layer of feeder fibroblasts 45. Such a co-culture system, involving key muscle resident non-myogenic cell types, would eventually allow for the ex vivo maintenance and genetic correction of isolated satellite cells without the dramatic loss of stemness that is observed in conventional culture systems.

## Cell-cell interactions in the satellite cell niche

Apart from the ECM and localized paracrine signals, cell-cell interactions are an important regulatory mechanism in the satellite cell niche. Satellite cells express several genes involved in cell-cell signaling at high levels. Amongst these are cadherins, neural cell adhesion molecule 1 (NCAM-1), vascular cell adhesion protein 1 (Vcam-1), intercellular adhesion molecule 1 (Icam1), claudin 5 (Cldn5), endothelial cell-specific adhesion molecule (Esam) 46. Moreover, Notch signaling which can be modulated by cell-associated and soluble ligands has been shown to be crucial for the maintenance of satellite cell quiescence 47, 48. However, the source of notch ligand in skeletal muscle has not yet been determined. Interestingly, Notch1 interacts with the HSPG Sdc3, and mice deficient for Sdc3 display impaired Notch signaling accompanied by a loss of satellite cell quiescence 49. These findings link the HSPG microenvironment to a key intracellular signaling pathway in satellite cells and are illustrative of the complexity that can arise from the interplay of niche components.

Recent evidence suggests that the elasticity of the niche is crucial for the function of satellite cells 23. The Blau laboratory demonstrated that soft hydrogel substrates that mimic the stiffness of muscle promote the engraftment of myogenic cells. By mimicking this physical characteristic of the niche in vitro, cells placed in the hydrogels maintained the expression of Pax7 better than those in plastic dishes. The main transplantation readout in this study was non-invasively measured by bioluminescence. However, it is difficult to distinguish whether the activity of this reporter is due to engraftment into the satellite cell niche or to differentiation and fusion to fibers. Future studies involving serial transplantation will hopefully resolve this question and address to which degree hydrogel culture promotes the maintenance of stemness and the self-renewal of cultured myogenic cells. Nevertheless, the work by Gilbert et al. shed light on the important fact that substrate elasticity can dramatically influence the character of myogenic cells.

Under homeostatic conditions, satellite cells are mostly quiescent remaining in a non-dividing G0 state 20. Following activation and tissue regeneration, these cells become dormant again. Although its exact purpose is not fully understood, quiescence in adult stem cells is considered a protective mechanism minimizing oxidative stress and preserving mitotic potential 50. Therefore, in a therapeutic setting, transplanted cells destined to sustainably repopulate the satellite cell compartment, ideally also have the capability to re-enter quiescence. Recent work has begun to elucidate the signaling pathways that the niche modulates to control whether the contained cells remain dormant or activated 51. The ability to promote satellite cell quiescence might have considerable potential for cell-based therapies. Maintenance of satellite cells in a quiescent state in vitro would likely prevent their terminal commitment and allow for the introduction of transgenes and subsequent autologous transplantation.

Taken together, the muscle stem cell niche integrates a plethora of molecular signals that control the maintenance of satellite cells in quiescence and their function upon activation 52. The instructive microenvironment in the niche is essential for self-renewal, commitment and differentiation of satellite cells during muscle regeneration. Based on our current knowledge, an inventory of this niche consists of an HSPG-rich extracellular microenvironment and its associated growth factors, fibroblastic and vessel associated cells, and specialized membrane domains on the muscle fiber. Cell-cell, cell-matrix contacts as well as signaling molecules released from local cells or systemic sources generate the major portion of extrinsic input for satellite cells and, in concert with intrinsic determinants, control their response to physiologic demands. A thorough understanding of the architecture of the muscle stem cell niche will hopefully pave the way for the development of biosynthetically optimized preservation and cultivation methods allowing for the genetic correction and/or expansion of satellite cells without a major loss of their stemness.

## Heterogeneity within the satellite cell pool

All quiescent satellite cells express the transcription factor paired-box 7 (Pax7) and some myogenic factor 5 (Myf5) 51. Upon activation most Pax7 positive satellite cells become myoblasts through upregulation of myoblast determination protein 1 (MyoD) 20. Eventually some of these myoblasts withdraw from the cell cycle and downregulate MyoD to return as reserve cells into quiescence 53, 54. However, the majority of MyoD expressing myoblasts differentiate by downregulating Pax7 and upregulating myogenin 55, 56. Finally, differentiated mononuclear myogenin and myosin heavy chain positive myocytes align and fuse to form multinucleated myotubes. Despite of accounting for only a few percent of the nuclear content of skeletal muscle, satellite cells are absolutely indispensable for tissue repair upon injury 57–60.

The satellite cell pool remains constant over multiple rounds of injury. Consequently, a self-renewing population of stem cells must exist within the myogenic progenitor pool. Early studies revealed that upon injury a subset of myogenic cells undergo immediate fusion into myofibers without preceding cell division while others, with delayed kinetics, enter mitosis 61. Subsequently, it has been shown that ∼20% of satellite cells accumulate DNA label at a slower rate than the rest of the myogenic pool 62. Moreover, a fraction of satellite cells distributes protein and chromatids in an asymmetric manner during mitosis 63–65. Along the same line of evidence, the transplantation of single freshly isolated satellite cells revealed that only a small percentage is able to engraft into the muscle stem cell niche 22. A caveat of this approach is that engraftment might not only reflect heterogeneity but could be due to stochastic survival of the cells. However, further evidence for the presence of progenitors with stem cell character within the satellite cell pool comes from studies investigating dystrophic and aged muscle. A subpopulation of satellite cells has been found to be resistant to high levels of radiation 66. Interestingly, this population of radiation resistant cells is exhausted in dystrophic, chronically de- and regenerating muscle. Moreover, only a fraction of atypical satellite cells appears to be resistant to aging 67. Taken together, these observations support the notion that separate populations of myogenic precursor cells exist within the satellite cell pool: committed satellite cells which are prone to differentiation and slower dividing satellite stem cells which maintain the myogenic progenitor pool by supplying daughter cells through asymmetric division.

A variety of different methodologies has been established to identify satellite cell subpopulations with stem cell character. The gold standard to test the stemness of a given myogenic subpopulation is transplantation into regenerating, eventually irradiated muscle with subsequent analysis of contribution of exogenous cells to the satellite cell compartment. This can be based on quantification of grafted cells in the satellite cell position on muscle sections or by fluorescence-activated cell sorting (FACS). Ideally, in such transplantation experiments, a given subpopulation of satellite cells with superior stemness is compared to the total pool of satellite cells or to the fraction of cells remaining after extraction of the stem population. Despite abundant reports on satellite cell heterogeneity only few studies have compared satellite cell subpopulations in this manner.

## Satellite stem cells

Based on expression reporter alleles and by immunostaining it has been shown that a fraction of satellite cells in adult muscle do not express Myf5 30, 68. Moreover, lineage tracing using a Myf5-Cre knock-in allele and an ROSA-YFP Cre reporter, revealed that about 10% of satellite cells have never expressed Myf5 in their developmental history 65. Upon activation, these Myf5 reporter negative cells self-renew by giving rise to Myf5/MyoD positive satellite cells through basal-apical oriented divisions or through planar symmetric divisions. The planar expansion of the Myf5 negative satellite cell pool is critically controlled by Wnt7a signaling and mice mutant for this factor display reduced numbers of satellite cells after muscle injury 69. Isolation of Myf5 reporter positive and negative cells followed by transplantation revealed that positive satellite cells preferentially differentiate, while negative satellite cells extensively contribute to the satellite cell compartment 65. Importantly, compared to Myf5 reporter positive cells, transplanted Myf5 reporter negative cells migrated large distances into the host muscle tissue.

As described by others 70, we observed that removal of Myf5 reporter negative satellite cells from their niche followed by in vitro culture converts this cell type into myoblasts capable of direct differentiation. Such myoblasts can differentiate into relatively pure Myf5 reporter negative myotubes, albeit these are smaller and formed with slower kinetics than those derived from normal myoblasts (our unpublished observation). These findings are consistent with the presence of muscle in Myf5 knockout mice and indicate that the self-renewal of Myf5 reporter negative cells through asymmetric division requires polarity input and/or other extrinsic cues from the satellite cell niche 69, 71. Myf5 knockout myogenic progenitors in the embryo show delayed differentiation kinetics that are, later in development, compensated for by unknown mechanisms. This observation likely reflects reduced myogenic commitment of Myf5 negative cells that is also found in postnatal muscle. In agreement with this hypothesis, it has recently been shown that satellite cells which are heterozygous for Myf5 have a higher capacity for self-renewal and niche occupancy after transplantation than wild-type cells 68. Taken together, these results demonstrate that satellite cells that do not express threshold levels of Myf5 are muscle stem cells that are required for the maintenance of the satellite cell pool.

In another line of research, Tanaka et al. characterized a subpopulation of satellite cells with characteristics of side population (SP) cells which express Pax7, Sdc3, Sdc4, ATP-binding cassette sub-family G member 2 (ABCG2) and stem cell antigen 1 (Sca1) 72. These satellite SP cells comprise about 3–10% of the satellite cell population and have a superior engraftment potential than total satellite cells in immunocompetent mice. Despite the fact that satellite SP cells cycle very slowly, their progeny can substantially contribute to the myonuclear compartment. This suggests that they undergo asymmetric division and that their more committed progeny can expand and differentiate to fuse with muscle fibers.

During regeneration, heterogeneous populations within satellite cells also behave differently in terms of proliferation kinetics. Asymmetric divisions giving rise to more differentiated progeny can be observed at almost every stage; satellite cell lineage progression thus allowing for a small population of satellite cells to retain stem cell characteristics. Based on a GFP live-reporter for Pax7 expression, Rocheteau et al. have shown that undifferentiated satellite cells with high Pax7 expression have the capability to asymmetrically distribute their chromatids when compared to cycling cells which have begun to downregulate Pax7 73. This study was first to compare a subpopulation of satellite cells that asymmetrically segregates chromosomes to another population with random segregation in a transplantation assay. Based on this experiment the authors concluded that the Pax7 high population has a higher engraftment potential than the Pax7 low population. This confirms the concept that cells with lower levels of Pax7 have a higher propensity for differentiation leading to their loss in serial transplantation. This study suggests that Pax7 high satellite cells are able to retain template strands of DNA after rounds of division. Such a mechanism is thought to protect stem cells from mutations arising from replication and allowing the retention of epigenetic marks such as DNA methylation. However, the mechanism by which this takes place is still under investigation. The work by Rocheteau et al. also raises the question whether the Pax7 high cells distributing their strands in an asymmetric manner are indeed a defined population or whether the satellite cell pool is in a state of flux with a higher probability for asymmetric division in the least differentiated cells.

It remains to be determined to which extent each of the above mentioned label and sorting approaches enrich for the same primitive “stem-like” population present in skeletal muscle and it is hoped that future studies will be able to unequivocally unravel the identity and biology of these satellite stem cells. Unfortunately, up to date no definitive stem cell marker has been established to isolate such satellite cells in species other than the mouse. The availability of a satellite stem cell marker that allows for sorting of living cells from human tissue would be of great use from a therapeutic point of view. It would allow for the selective enrichment of these cells from donor tissue, reduce immunogenicity due to the low number of cells required for transplantation, decrease the number of injections required to obtain a therapeutic effect and it would result in a sustained muscle-wide repopulation of the host stem cell niche by donor cells without loss due to excessive differentiation. Another problem associated with the intramuscular delivery of myogenic cells that could be solved by transplantation of few stem cells is the massive cell death that has been described upon grafting of large amounts of myoblasts into muscle 74–76. It has been shown that the survival of transplanted cells depends on the number of cells and the volume of injection vehicle, which can lead to the formation of large intramuscular injection pockets causing inefficient nutrient supply and ischemic necrosis. Moreover, given the ability to maintain these cells long enough ex vivo for their genetic correction and autologous transplantation, grafting satellite stem cells would likely result in a life-long therapeutic effect.

## Non-satellite cell types with myogenic potential

A variety of cells different from satellite cells possess myogenic potential (Table 1). Some of these atypical myogenic cell types are considered as potential therapeutics for muscular dystrophy. Most promising amongst these are mesoangioblast-like cells/pericytes. These cells are perivascular mesenchymal-like progenitors that can differentiate into various cell types of mesodermal origin, including skeletal muscle fibers and cardiac muscle 77, 78. Such cells have been isolated from embryonic and postnatal aorta, bone marrow, cardiac, and skeletal muscle, as well as other tissues 79. The ease of transduction with viral vectors and the ability of these cells to cross the endothelial wall in the presence of inflammation, as in the case of muscular dystrophy, makes them very interesting therapeutic candidates for systemic delivery 80. Recent reports revealed that human pericytes as well as genetically corrected dystrophin deficient murine pericytes can not only fuse to muscle fibers but generate cells in the satellite cell position 81, 82. Taken together, this atypical myogenic cell type holds outstanding therapeutic promise and translational potential for the treatment of muscular dystrophy.

**Table 1:** Known advantages and problems associated with the possible therapeutic use of different myogenic cell types

Cell type	Advantages	Major problems	Ref.
Myoblasts	• Pure populations can be isolated and readily expanded and transduced in vitro	• Limited engraftment and migration in host muscle	8, 24, 100
		• High number of cells required for transplantation	
		• Immediate immune response after grafting due to high number of cells	
		• Poor ability to populate the host satellite cell niche	
Satellite cells	• Low numbers required for transplantation	• Limited migration	21, 22, 101, 102
	• Efficient engraftment	• Only small numbers can be isolated	
	• Efficient population of the satellite cell niche of the recipient	• Cannot be cultured/maintained ex vivo	
Satellite stem cells	• Very efficient engraftment	• No definitive markers available for the enrichment of viable cells	65, 68, 73
	• Few cells required for transplantation		
	• Highly efficient population of the satellite cell niche of the recipient	• Not investigated in species other than mouse	
	• Extensive migration		
Satellite cells on fibers	• Maximal engraftment	• Very difficult to apply in a clinical setting	25, 36, 65
	• Minimal number of cells required		
	• Maximal population of the satellite cell niche		
Muscle side population cells	• Certain SP cells can home into muscle from the blood stream (systemic delivery possible)	• Contact with myoblasts required for differentiation	90, 103, 104
	• Population of the satellite cell niche of the recipient	• Low engraftment	
Mesoangioblasts/pericytes	• Homing from the blood stream into the muscle (systemic delivery possible)	• Undergo senescence after a certain number of population doublings	81, 82, 105–107
	• Can be cultivated ex vivo		
	• Readily transducible		
		• Sufficient engraftment	
	• Engraftment as satellite cells		
CD133 positive cells	• Homing from the blood stream into the muscle (systemic delivery possible)	• Engraftment only shown in animal models with severely compromised immune system	85, 86
	• Increased vasculogenesis		
	• Engraftment as satellite cells		
Myoendothelial cells	• Can be cultured for a long period retaining myogenic potential	• Engraftment only shown in animal models with severely compromised immune system	87
	• Tolerance for oxidative stress		
Muscle resident ALDH positive CD34 negative cells	• High proliferative potential upon transplantation	• Engraftment only shown in animal models with severely compromised immune system	88
PW1+ interstitial cells	• Engraftment as satellite cells	• Only shown in a mouse model with severely compromised immune system	89
Bone marrow derived stem cells	• Homing from the blood stream into the muscle (systemic delivery possible)	• Low engraftment	91, 108
Mesenchymal stem cells	• Inhibition of inflammation	• Low engraftment	93, 94
hMAD: human mesenchymal stem cell from adipose tissue	• Easy to access from adipose tissue	• Low engraftment potential without forced expression of MyoD	109, 110
	• Engraftment only shown in animal models with severely compromised immune system		
ES cells	• Engraftment as satellite cells	• Risk of teratoma formation	97, 111
		• Pax3/7 overexpression required for reprogramming	
iPS cells	• Engraftment as satellite cells	• Risk of teratoma formation	112
	• Autologous transplantations possible	• Reprogramming and purification required	
		• Differentiation may be impaired by epigenetic memory of the donor tissue	

Several other cell types with varying myogenic potential that could be of therapeutic relevance have been described. (i) A rare population of cells expressing CD133 or Prominin-1 that is present in human skeletal muscle and circulating in adult blood has myogenic potential 83–85. In co-culture with myogenic cells, CD133 positive progenitors differentiate into myotubes. Upon intramuscular application or injection into the bloodstream these cells are able to home to the satellite cell niche and to generate fibers expressing human dystrophin in immunocompromised mdx mice more efficiently than human myoblasts 85. Interestingly, local injection of this cell type seems to promote muscle regeneration through increased vasculogenesis 86. These studies suggest that the systemic delivery of CD133 positive cells from immunologically matched healthy donors or genetically corrected cells from patient blood could be a feasible strategy for the treatment of muscular dystrophy. (ii) In human muscles a population of myoendothelial cells that co-express myogenic and endothelial cell markers (CD56, CD34, CD144) have been described to extensively contribute to regeneration upon transplantation into cardiotoxin injured skeletal muscle of SCID mice 87. Interestingly, this cell type can be cultured clonally for long periods while retaining its myogenic properties. (iii) Another population of muscle resident cells has been shown to express aldehyde dehydrogenase (ALDH) but not CD34 88. These cells can participate in muscle formation and populate the satellite cell compartment upon injection into injured muscle of immunocompromised mice. ALDH positive and CD34 negative cells appear to have an outstanding capacity for proliferation upon transplantation. (iv) The Sassoon laboratory has discovered muscle resident PW1+ interstitial cells (PICs) which possess Pax7 dependent myogenic activity during postnatal muscle growth, contribute to skeletal muscle regeneration and are able to generate satellite cells 89. (v) Asakura et al. have described a fraction of Sca-1 positive SP cells found in muscle which undergo myogenic specification after co-culture with myoblasts 90. If injected into regenerating muscle of SCID mice, SP cells give rise to both myocytes and satellite cells. (vi) Several groups have shown that bone marrow-derived cells, such as hematopoietic and mesenchymal stem cells, can participate in the regeneration of muscle, albeit with a very low efficiency 91–95. (vii) Last but not least, embryonic (ES) and induced pluripotent (iPS) stem cells are currently explored as potential candidates for cell therapy of muscular diseases. Barberi et al. were able to derive engraftable myoblasts from human ES cells 96. Gene therapies with patient derived corrected iPS cells offer an advantage over ES cells by providing a genetic match and thereby decreasing the likelihood of immunorejection. Darabi et al. reported the generation of functional skeletal muscle from mouse ES and iPS cells by ectopic expression of Pax3/7 and the engraftment of these cells into the satellite cell niche in dystrophic mice 97, 98. Because of the possible development of teratomas, the use of ES and iPS cells will have to be carefully monitored in a clinical setting 99.

## A basic research perspective: Current status and future directions

So far, a wide variety of cells with myogenic potential harboring distinct advantages and disadvantages for therapy have been discovered. In the recent years, basic research has begun to define parameters for experimental cell-based treatments that are likely to be translationally relevant. Desirable characteristics of transplanted cells are a high proliferative capacity without tumorigenicity, a minimal immunogenicity, the ability to cross the blood-muscle barrier and/or a robust migratory capacity within muscle tissue, the ability to sustainably repopulate the satellite cell niche, the ability to return to quiescence, and an unimpaired differentiation potential allowing for an extensive transfer of genetic material into muscle fibers. Apart from isolating sufficient cells with these characteristics, some of the main limitations we are facing now are the lack of ex vivo cultivation methods allowing for genetic correction while preserving satellite cell “stemness”, the lack of definite markers for the enrichment of viable satellite stem cell populations, inefficient delivery methods and a lack of strategies to efficiently control fibrosis and inflammation. At present, the ability to cross the blood-muscle barrier after systemic delivery and the capability to populate the satellite cell niche makes mesoangioblast-like cells/pericytes the most promising candidates for cell-based therapy of muscular dystrophy. Future research will have to advance the feasibility of alternative approaches such as the genetic correction and/or grafting of satellite cells so they can be assessed for their efficiency in a clinical setting.

## Conclusions

It is now more than 50 years since the satellite cell of skeletal muscle has been discovered 29. Ever since a wide field of research has evolved around it and fundamental insights into the fascinating nature of these cells have been obtained. The concept of hierarchical heterogeneity within the satellite cell pool and the muscle stem cell niche as a dynamic entity is now widely accepted and awaits translational research beyond the mouse-model system. The potential existence of a “true” satellite stem cell in human muscle is an exciting possibility that could be explored in multiple ways for the treatment of degenerative muscular diseases. Ongoing research will help to further dissect the niche components controlling the stemness of satellite cells with the ultimate goal to design biological substrates allowing for the ex vivo maintenance and genetic correction of satellite cells. Apart from that, recent important discoveries such as atypical myogenic progenitors that are suitable for systemic delivery or the generation of iPS cells that can be programmed to engraft as satellite cells hold great therapeutic promise. Unfortunately, the path from basic scientific discovery to methods and technologies that can be applied in a clinical setting is lengthy and complicated. It is hoped that in the future, translational success will be fostered and accelerated by innovative research networks bringing together academia, industry, and clinicians.

## Abbreviations

ALDH: aldehyde dehydrogenase
bFGF: basic fibroblast growth factor
ECM: extracellular matrix
ES: embryonic stem
FACS: fluorescence-activated cell sorting
FAP: fibro/adipogenic progenitor
HSPG: heparan sulfate proteoglycans
iPS: induced pluripotent stem cells
MMP: matrix metalloproteinases
SP: side population
VEGF: vascular endothelial growth factor.
